# Enhanced Homing Technique of Mesenchymal Stem Cells Using Iron Oxide Nanoparticles by Magnetic Attraction in Olfactory-Injured Mouse Models

**DOI:** 10.3390/ijms19051376

**Published:** 2018-05-05

**Authors:** Wan Su Yun, Jin Sil Choi, Hyun Mi Ju, Min Hee Kim, Seong Jin Choi, Eun Seol Oh, Young Joon Seo, Jaehong Key

**Affiliations:** 1 Department of Biomedical Engineering, Yonsei University, Wonju, Gangwon-do 26493, Korea; ip9801@naver.com (W.S.Y.); marchman10@hanmail.net (E.S.O.); 2 Laboratory of Smile Snail, Yonsei University Wonju College of Medicine, Wonju, Gangwon-do 26426, Korea; towtowtow92@naver.com (J.S.C.); skdi1082@naver.com (H.M.J.); mini.aclice11234@gmail.com (M.H.K.); 3 Department of Otorhinolaryngology, Yonsei University Wonju College of Medicine, Wonju, Gangwon-do 26426, Korea; 4 Department of Obstetrics and Gynecology, Yonsei University Wonju College of Medicine, Wonju, Gangwon-do 26426, Korea; choisj@yonsei.ac.kr

**Keywords:** superparamagnetic iron oxide nanoparticles, CXCR4, homing, mesenchymal stem cells, intranasal delivery, olfactory-injured mouse model

## Abstract

Intranasal delivery of mesenchymal stem cells (MSCs) to the olfactory bulb is a promising approach for treating olfactory injury. Additionally, using the homing phenomenon of MSCs may be clinically applicable for developing therapeutic cell carriers. Herein, using superparamagnetic iron oxide nanoparticles (SPIONs) and a permanent magnet, we demonstrated an enhanced homing effect in an olfactory model. Superparamagnetic iron oxide nanoparticles with rhodamine B (IRBs) had a diameter of 5.22 ± 0.9 nm and ζ-potential of +15.2 ± 0.3 mV. IRB concentration of 15 µg/mL was injected with SPIONs into MSCs, as cell viability significantly decreased when 20 μg/mL was used (*p* ≤ 0.005) compared to in controls. The cells exhibited magnetic attraction in vitro. SPIONs also stimulated CXCR4 (C-X-C chemokine receptor type 4) expression and CXCR4-SDF-1 (Stromal cell-derived factor 1) signaling in MSCs. After injecting magnetized MSCs, these cells were detected in the damaged olfactory bulb one week after injury on one side, and there was a significant increase compared to when non-magnetized MSCs were injected. Our results suggest that SPIONs-labeled MSCs migrated to injured olfactory tissue through guidance with a permanent magnet, resulting in better homing effects of MSCs in vivo, and that iron oxide nanoparticles can be used for internalization, various biological applications, and regenerative studies.

## 1. Introduction

Stem cell-based therapy is actively studied and used in all areas of regenerative medicine. However, delivery of an appropriate number of intact cells to defective tissue remains difficult. Stem cells have self-renewal capability and can differentiate into various tissues [1]. In addition, stem cell migration to a damaged cell, known as the homing phenomenon, is an important part of stem cell research. Human mesenchymal stem cells (MSCs) communicate with other cells in the body and appear to ‘home’ to injured tissue in response to cellular damage signals known as homing factors [2].

Homing may be useful in clinically applying MSCs as cell carriers for therapeutic modalities. MSCs injected either topically or systematically have been used for cell therapy for various indications. Bone marrow MSCs are used to alleviate clinical symptoms of incomplete bone formation and infarcted myocardium [3,4] and been applied as immunomodulatory treatments for autoimmune diseases including Crohn’s disease [5], multiple sclerosis [6], and rheumatoid arthritis [7]. Homing may be clinically applied with MSCs as cellular mediators for anti-cancer therapy in tumors. Maestroni et al. [8] showed that bone marrow MSCs significantly reduced the size and metastasis of lung cancer cells and melanoma cells in mice. Although the olfactory epithelium can regenerate continuously, few studies have examined restoration of the olfactory epithelium using stem cell techniques [9].

Although the precise mechanism of how MSCs select their target tissues is not completely understood, several previous studies suggested that chemokines and their receptors (e.g., CXCR4 and SDF-1) are important factors that induce homing of MSCs. To improve the effectiveness of MSC homing, several strategies have been developed. (1) Cultivate MSCs to have a higher migratory capability by adjusting the cell culture conditions. Shi et al. [10] showed that MSCs with a cocktail of cytokines in culture induced high surface expression of CXCR4, with chemotactic receptors of SDF-1α up-regulated in ischemic tissues. (2) Improve the capability of MSCs to respond to migratory stimuli. Several studies to modify MSCs or increase the expression of surface markers (CXCR4-SDF-1α axis) have been conducted to improve MSC migration. (3) Stimulate the target site for MSC mobilization. François et al. [11] applied whole body irradiation or additional local irradiation to the abdominal area or hindlimb of mice.

Recently, a “magnetic attraction” method for stem cells was developed. Two previous studies evaluated the magnetic attraction of stem cells to the brain. Song et al. [12] demonstrated that rats wearing an external magnet (0.32 T) on their skull for one week contained an increased number of stem cells labeled by superparamagnetic iron oxide (SPIO) after intravenous injection, resulting in a 3-fold or higher increase in the infarct area under the magnet as well as a significant decrease in the infarct size. Shen et al. [13] introduced another approach for magnetic stem cell attraction to injury sites after traumatic brain injury via intra-carotid delivery.

**Scheme 1:**
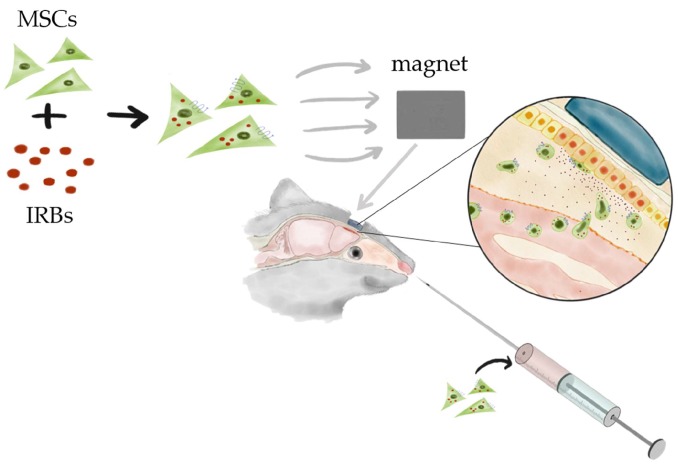
Schematic illustration of mesenchymal stem cell (MSC) homing in olfactory mouse model using a permanent magnet. Iron oxide nanoparticles internalized in MSCs guide the cells to the defective site using an external permanent magnet.

SPIOs are known to induce reactive oxygen species (ROS) [14,15], and ROS increases CXCR4 expression of MSCs derived from bone marrow [16]. Huang et al. demonstrated that internalization of SPIOs into MSCs strengthens the CXCR4-SDF-1α axis in a Transwell assay [17]. Taken together, our results show that magnetic retention of SPIO-labeled MSCs increased the migration and homing efficiency of MSCs with SPIO nanoparticles in vivo in mice with olfactory bulb injury (Scheme 1).

## 2. Results

### 2.1. Characterization of Nanoparticles

**Figure 1:**
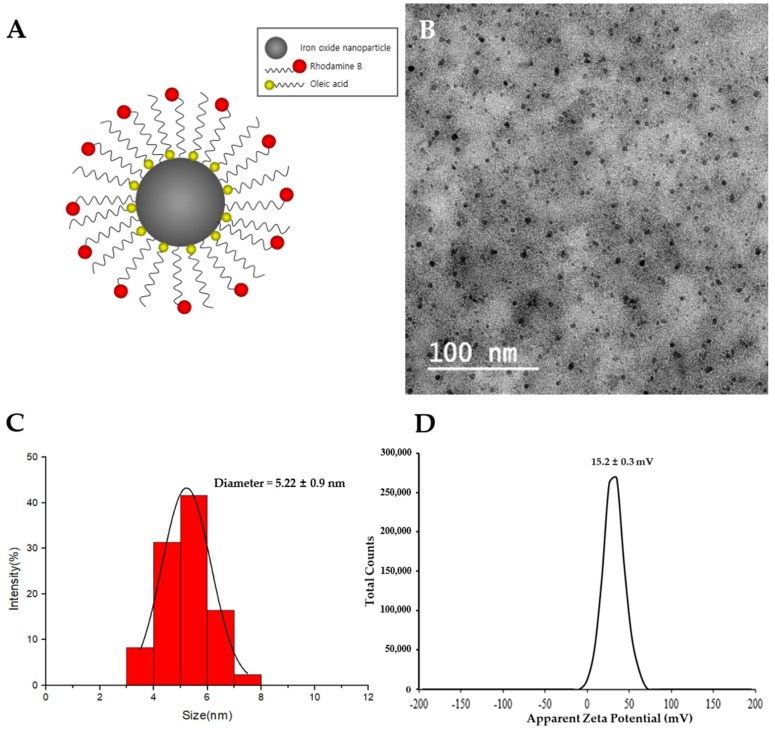
Characterization of superparamagnetic iron oxide (SPIO) nanoparticles with rhodamine B (IRBs). (**A**) Schematic illustration of IRBs used in this study. (**B**) Transmission electron microscope image of mono-dispersed IRBs. (**C**) Histogram analysis of the diameter of IRBs in transmission electron microscopy (TEM) images. (**D**) ζ-potential results of IRBs (*n* = 3) using Zetasizer-ZS90.

The proposed SPIO nanoparticles with rhodamine B (IRBs) were comprised of an SPIO core coated with both oleic acid and rhodamine b, which were purchased from Ocean NanoTech (Springdale, AR, USA) [18] (Figure 1A). The sizes of IRBs were measured by transmission electron microscopy (TEM). A Zetasizer-ZS90 was utilized to measure the ζ-potential. TEM images showed that IRBs appeared as uniform spheres under completely dried conditions (Figure 1B). To analyze the IRB diameter, ImageJ software was used (NIH, Bethesda, MD, USA). After randomly sampling 255 particles, the IRB diameter was found to be 5.22 ± 0.9 nm (Figure 1C). The ζ-potential of IRBs was slightly positive in aqueous solution with a value of +15.2 ± 0.3 mV (Figure 1D).

### 2.2. Internalization of IRBs (SPIO nanoparticles with rhodamine b) into MSCs (Mesenchymal stem cells) and Magnetic Properties

**Figure 2:**
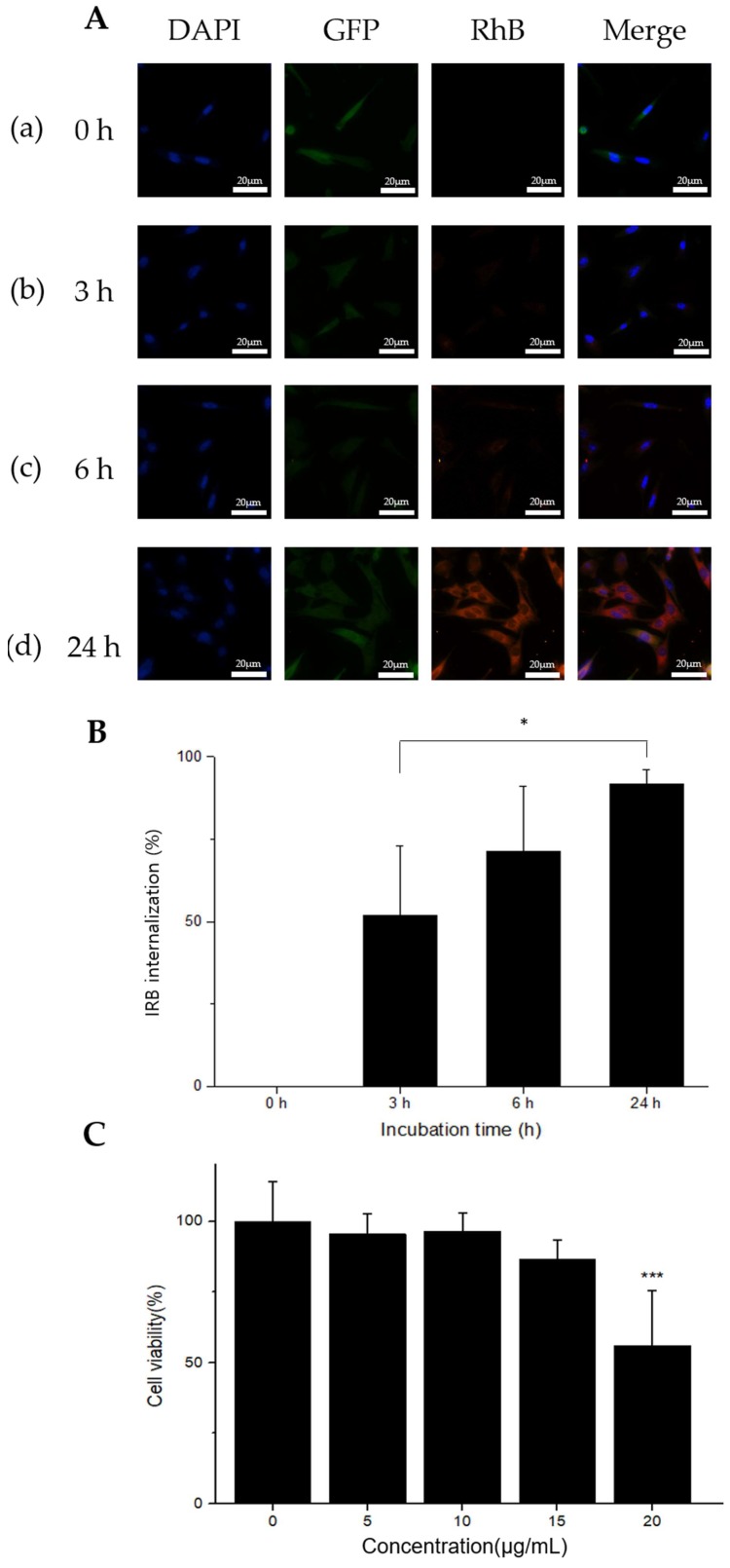
Cell internalization and viability analysis by IRBs. (**A**) Fluorescence microscopy images confirming IRB uptake at different incubation times. Each experimental group was incubated for 24 h. Time points of 0 h (**a**), 3 h (**b**), 6 h (**c**), and 24 h (**d**) in 15 μg/mL of IRBs (60× magnification, scale bar: 20 μm). (**B**) Ratio of IRB internalization in MSCs. IRB internalization was 0% at 0 h, 52% at 3 h, 71.4% at 6 h, and 91.6% at 24 h (*n* = 4, *: *p* ≤ 0.05). (**C**) In vitro CCK-8 cytotoxicity analysis of IRBs in MSCs. Results presented as cell viability (mean ± SD) versus IRB concentration. Viability results were normalized to the control groups (*n* = 9, ***: *p* ≤ 0.005).

Cellular internalization of IRBs was characterized by measuring the red fluorescence of rhodamine B-labeled IRBs (Figure 2A). Green fluorescence indicated green fluorescent protein (GFP)-labeled MSCs. MSC nuclei were stained with 4′,6-diamidino-2-phenylindole (DAPI). MSCs in each image (Figure 2A (a)–(d)) were treated and incubated for 0, 3, 6, and 24 h with 15 μg/mL IRBs. Significant differences were observed in each image. With increasing incubation time, a greater number of IRBs gradually became internalized into the MSCs as measured at 580 nm. Thus, the group treated for 24 h with IRBs showed the largest number of IRBs in the MSCs. The ratio of IRB internalization in MSCs was measured with a fluorescence microscope (Figure 2B). The internalization ratios were 0% at 0 h, 52% at 3 h, 71.4% at 6 h, and 91.6% at 24 h. The results showed that as incubation time increased, the internalization percent also increased. Therefore, for sufficient internalization, 24-h IRB incubation was selected.

The in vitro cytotoxicity of IRBs was measured by the (2-(2-methoxy-4-nitrophenyl)-3-(4-nitrophenyl)-5-(2,4-disulfophenyl)-2*H*-tetrazolium) (CCK-8) assay (Figure 2C). Cell viability in 20 μg/mL samples was significantly decreased (*p* ≤ 0.005) compared to that in control samples. The results indicated that the highest concentration was considerably cytotoxic. Thus, 15 μg/mL of IRB was used for CXCR4 expression and further in vivo evaluation.

**Figure 3:**
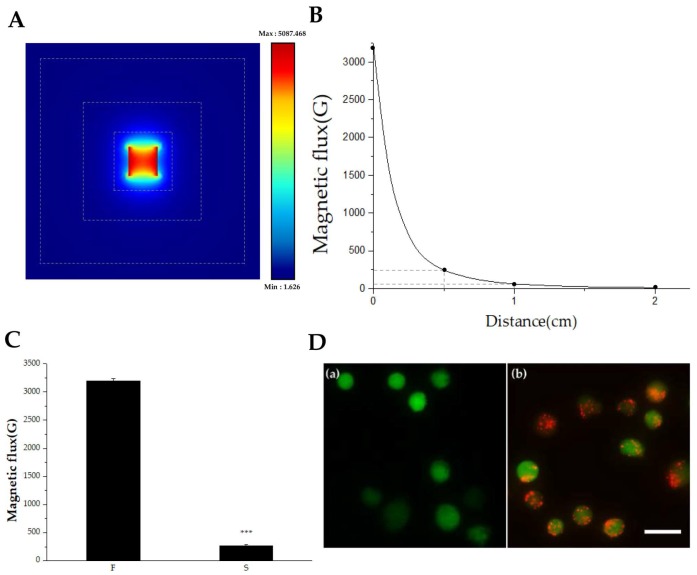
Measurement of magnetic flux and visualization of IRB-loaded MSCs. (**A**) Simulation analysis of a permanent cube-shape magnet. The dotted lines indicate the distances from the magnetic surface, which were 5, 10, and 20 mm. Different magnetic flux values depending on distances and directions. (**B**) Graph of magnetic flux versus distance. The actual values of magnetic flux from a permanent magnet applied in this study were measured. Magnetic flux values were 3200, 235, 60, and 10 Gauss at 0, 5, 10, and 20 mm, marked as a black dot, respectively. (**C**) Different surface magnetic flux was measured depending in polar (F) and non-polar directions (S) (*n* = 9, ***: *p* ≤ 0.005). (**D**) IRB-loaded MSCs floating in the cell culture media were visualized with a fluorescence microscope, showing a clear difference between (**a**) MSCs without IRBs and (**b**) IRB-loaded MSCs (Scale bar: 25 μm).

For magnet attraction experiments, magnetic flux and effective distances were tested. The permanent magnet used in this study was analyzed by both COMSOL (Burlington, MA, USA) simulation and a magnetometer. Figure 3A shows the simulation results. Magnetic flux was evaluated from a permanent cube-shape magnet, which revealed differences in magnetic flux depending on both the direction and distance. The maximum magnetic flux was 5087 Gauss and minimum flux was 1.626 Gauss. The result showed that the north and south magnetic fluxes changed symmetrically with distance. However, only very weak magnetic fluxes were observed on the sides. Therefore, only the north and south poles were evaluated by magnetometer analysis. To analyze the differences between the polar surface and non-polar surface, magnetic flux was measured with a magnetometer (KANETEC, Tokyo, Japan) at 1-mm intervals. The result showed that magnetic flux decreased significantly as the distance from the magnet increased (Figure 3B). Surface magnetic flux at the north pole was 0.32 T (3200 Gauss) and flux at the non-pole was nearly 0.03 T (264 Gauss) (Figure 3C). Thus, only the polar surface was considered for further experiments, considering the effective distance from the surface (*n* = 9, *p* ≤ 0.005).

It is important to evaluate whether MSCs hold IRBs under the floating condition because the MSCs were injected into the bloodstream and physically dragged to the injured area via an external magnet. To confirm that IRBs were internalized into MSCs under floating conditions, MSCs were incubated with IRBs at 37 °C for 24 h and treated with trypsin for 2 min. MSCs were observed by fluorescence microscopy (Figure 3D). In contrast to MSCs without IRBs (Figure 3D (a)), MSCs with IRBs clearly showed internalized IRBs even in the floating state (Figure 3D (b)). 

### 2.3. Enhanced Migration Capacity of Magnetized MSCs with IRBs In Vitro

**Figure 4:**
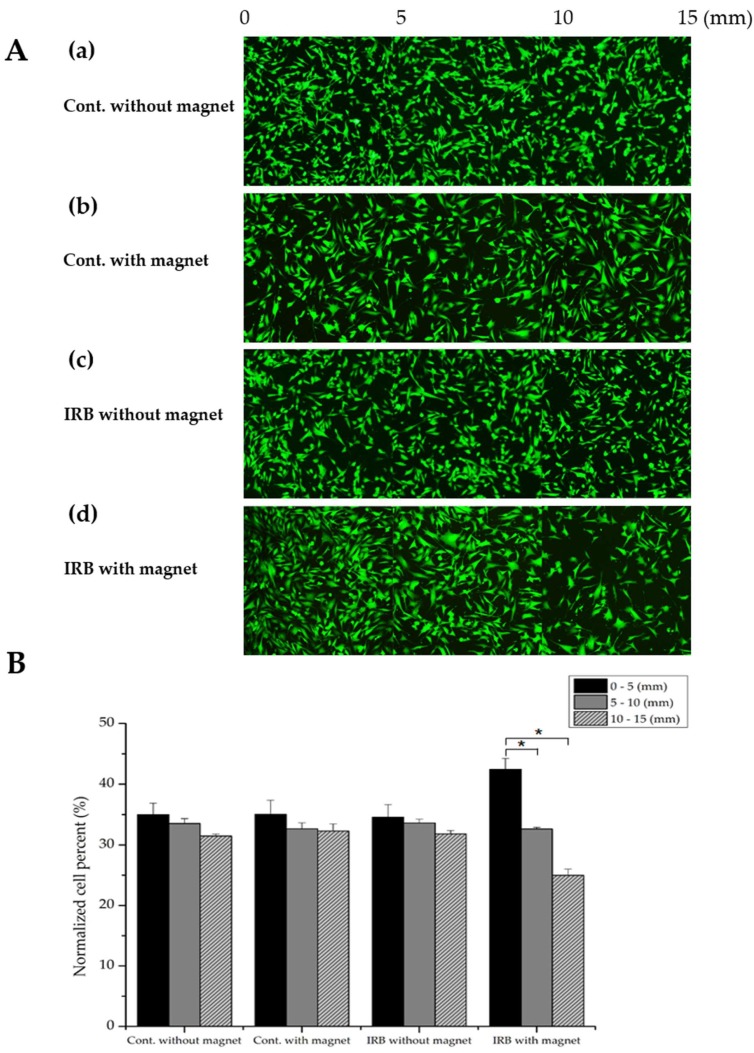
Visualization and quantitative analysis of magnetic dragging of MSCs. (**A**) (**a**)–(**c**): MSCs only, MSCs with a magnet, and IRB-loaded MSCs without a magnet showed no magnetic dragging. (**d**): IRB-loaded MSCs clearly showed magnetic dragging. (**B**) Normalized cell counting of MSCs depending on the distance from the magnet showed that only IRB-loaded MSCs were attracted by the magnet (approximately 43% of MSCs at 0–5 mm) (*n* = 4, *: *p* ≤ 0.05).

Figure 4A shows that MSCs with IRBs were magnetically attracted. The fluorescence intensity of GFP-tagged MSCs was determined, and a normalized number of MSCs affected by the magnet was analyzed with ImageJ software (Figure 4B). MSCs without IRBs and 15 μg/mL magnetized MSCs were prepared in 6-well plates. After 24 h of incubation, fluorescence images were acquired at up to 15 mm at 1-mm intervals from the magnet (Figure 4A (a)–(b)). The cells were treated with trypsin and seeded into 6-well plates, which were attached by a magnet outside the wall. After 24 h of incubation, fluorescence images were captured using the same steps (Figure 4A (c)–(d)). In the plate without the magnet, 34.99% of cells without IRBs adhered in the range of 0–5 mm (front part), 33.54% adhered in 5–10 mm (middle part), and 31.46% adhered to 10–15 mm (back part); 35.02% of magnetized MSCs adhered to the front part, 32.68% adhered to the middle part, and 32.29% adhered to the back part. In the plate with a magnet, 34.57% of MSCs without IRBs adhered to the front part, 33.60% adhered to the middle part, and 31.82% adhered to the back part. In contrast, 42.42% of magnetized MSCs adhered to the front part, 32.61% adhered to the middle part, and 24.97% adhered to the back part (Figure 4B). As a result, only in the group treated with IRBs with an external magnet, MSCs were effectively attracted to the area showing the magnetic force because of magnetic flux. These results revealed magnetized MSC migration in vitro using a permanent magnet.

### 2.4. Enhanced Expression of CXCR4 in Magnetized MSCs with IRBs and Reactive Oxygen Species (ROS) Analysis

**Figure 5:**
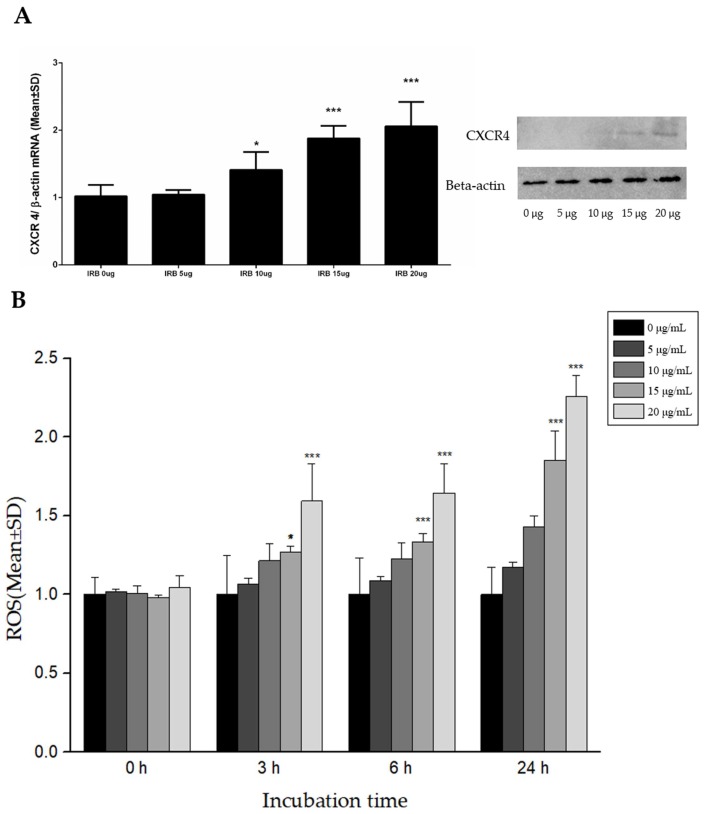
Expression of CXCR4 by Western blotting and measurement of reactive oxygen species (ROS) by H_2_DCFDA using fluorescence intensity. (**A**) Different IRB concentrations induced different expression levels of CXCR4. At a concentration of 15 μg/mL, CXCR4 RNA was increased by approximately 2-fold (*n* = 8, *: *p* ≤ 0.05, ***: *p* ≤ 0.005). (**B**) Different IRB concentrations and IRB incubation time induced changes in ROS levels. After 3 h, 20 μg/mL IRBs clearly generated ROS compared to 0 μg/mL. After 24 h, 15 μg/mL induced a nearly 2-fold increase in ROS levels compared to in the control and 20 μg/mL induced a more than 2-fold increase in ROS levels compared to in the control. H_2_DCFDA and IRBs were added to each well at the same time (*n* = 9, *: *p* ≤ 0.05, ***: *p* ≤ 0.005).

CXCR4 expression in cells was increased by internalizing IRBs into the cells (Figure 5A). As the concentration of IRB increased, the amount of CXCR4 RNA was significantly increased by 10 μg/mL IRB compared to the control, which was increased by approximately 2-fold (*p* ≤ 0.005) at an IRB concentration of 15 μg/mL. This pattern confirmed the results of protein quantification obtained by western blotting.

The relationship between ROS levels in magnetized MSCs and IRBs was evaluated (Figure 5B). ROS were measured as the normalized intensity of 2′,7′-dichlorodihydrofluorescein diacetate (H_2_DCFDA) fluorescence. Immediately after IRBs were added to the MSCs, there were no differences among IRBs concentrations. However, after 3, 6, and 24 h of incubation, ROS values increased proportionally with IRB concentration. Changes in ROS levels were related to CXCR4 expression levels at 24 h. At 15 μg/mL IRBs at 24 h, the levels of ROS in MSCs were significantly increased by nearly 2-fold (*p* ≤ 0.005) compared to in the control.

### 2.5. Enhanced Migration of Magnetized MSCs with IRBs In Vivo in Olfactory-Injured Mouse Models

Preparation of olfactory-injured mouse models and migration of magnetized MSCs with IRBs in mice were performed (Figure S1). To prepare olfactory-inured mouse models, the scalp bone was exposed by cutting the skin covering the skull with scissors. A 2-mm cranial window was opened on the exposed bone. Bipolar coagulation was used with a 1-mm depth for 100 ms on only one side of the olfactory bulb to induce olfactory injury. The result was confirmed by H&E (Hematoxylin and Eosin) staining and immunohistochemistry (Figure S2). The 5-mm cuboidal magnet was inserted into the scalp, and then the scalp was sutured. Injection of magnetized MSCs incubated with 15 μg/mL IRBs for 24 h into the olfactory-injured mouse model was performed with a 50-μL Hamilton syringe via each nostril (Supplementary Movie 1).

**Figure 6:**
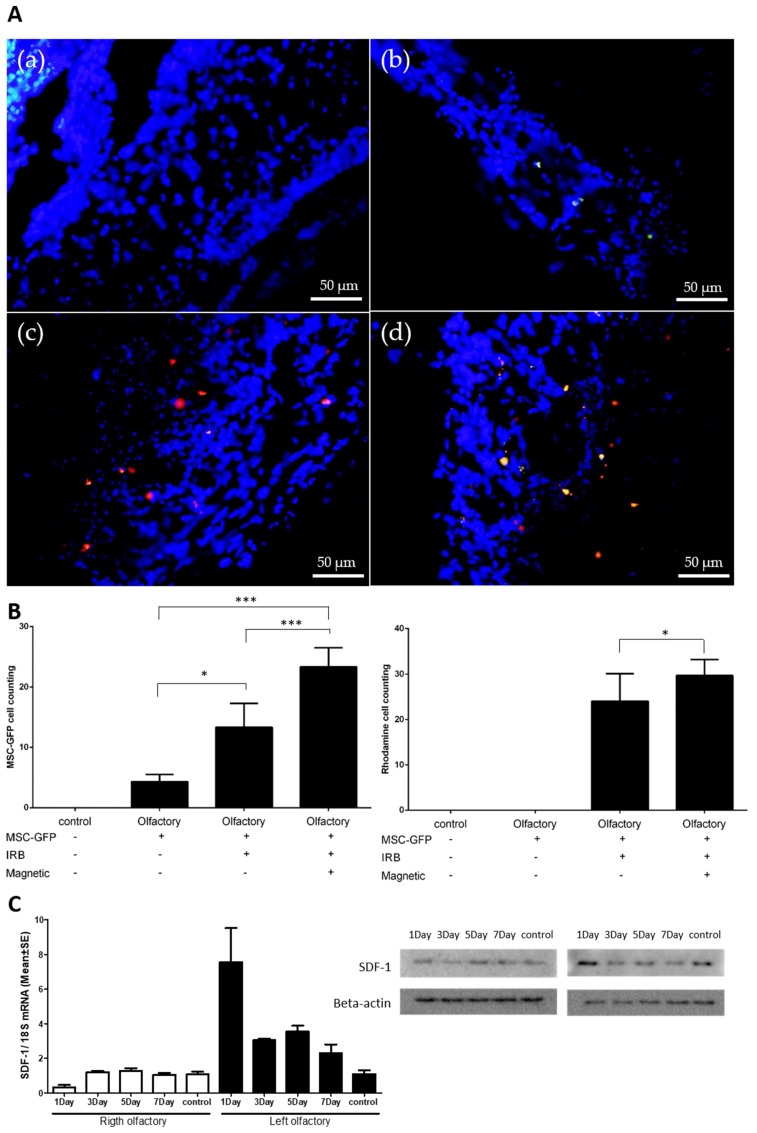
Enhanced migration of magnetized MSCs with IRBs in olfactory-injured mouse models. Green dots—GFP-tagged MSCs, Red dots—rhodamine-tagged (IRB) MSCs. (**A**) (**a**) control: injection without MSCs in normal mice, (**b**) MSC injection without IRBs, (**c**) magnetized MSC injection with IRBs, (**d**) magnetized MSC injection with IRBs under magnetic field (scale bar: 50 μm). (**B**) Graphs of MSC-GRP cell counting (**left**) and rhodamine cell counting (**right**) according to injection groups (*n* = 8, *: *p* ≤ 0.05, ***: *p* ≤ 0.005). (**C**) Increased expression of SDF-1 in injured olfactory bulb (**left**) determined by PCR and Western blotting (**right**) (control *n* = 4, experimental group *n* = 12).

One week after MSC injection, the olfactory bulb was extracted to confirm the presence of stem cells in the damaged olfactory bulb. We compared the presence of MSCs in the damaged olfactory bulb under four different conditions: (a) control without MSC injection, (b) MSC injection without IRBs, (c) MSC injection with IRBs, (d) MSC injection with IRBs under magnetic fields. (a) Control showed neither GFP nor rhodamine signals in the injured area. (b) MSC injection without IRBs showed few GFP signals in the area. Only (c) and (d) showed both GFP and rhodamine signals (Figure 6A).

To quantify the results, we counted the number of MSCs in the injured area using both GFP signals from MSCs and rhodamine signals from IRBs in MSCs (Figure 6B). For counting by GFP, MSC injection with IRBs under a magnetic field showed significantly higher values compared to both MSC injection without IRBs and MSC injection with IRBs (*p* ≤ 0.005). (Figure 6B, Left). For counting by rhodamine, MSC injection with IRBs under a magnetic field also showed a meaningful difference compared to MSC injection without IRBs (*p* ≤ 0.05) (Figure 6B, Right). The different values between GFP and rhodamine counting may be explained by the optical sensitivity of the microscope used.

SDF-1 RNA and protein levels showed the greatest increase at 1 day after injury but remained higher than in the control after seven days (Figure 6C). This may explain why MSC injection with IRBs without magnetic fields showed higher homing effects than MSCs alone (*p* ≤ 0.05) (Figure 6B-Left). Increased CXCR4 levels in the MSCs via the internalization of IRBs may improve the CXCR4-SDF-1 signaling pathway at the injured area (Figure 5A).

## 3. Discussion

Our study reveals methods for improving the homing ability of magnetized MSCs with internalized IRBs in vivo. By internalizing the IRBs, the direct homing efficiency of MSCs to the wounded olfactory bulb was improved by promoting the homing factor of the CXCR4-SDF-1 axis, and additional MSCs were be homed to the desired site by using a magnetic field. Although IRB internalization may affect MSC proliferation or vitality at high doses, an appropriate amount of IRB did not inhibit the cells, but rather had a stimulatory effect, leading to increased ROS because of the hypoxic conditions, which may help CXCR4 to increase the homing of MSCs. A sufficient number of IRBs was internalized into the cell and responded to the magnetic field, which was useful for promoting MSC homing in vivo. Previous studies also introduced nanoparticles to enhance homing. Magnetic attraction of SPIO-labeled cells has been applied to enhance the delivery of stem cells to a wide range of target tissues including the liver, muscle, joints, heart, retina, and brain [13,15,19,20,21]. Studies observed increased cells in the target tissue as well as physiological improvement. Shen et al. [13] transported human SPIO-labeled neuroprogenitor cells (hNPCs) into post-traumatic brain injury animals in the presence of a static magnetic field. They revealed increased homing and retention of hNPCs to the injured cortex compared to in the control group in which hNPCs were injected in the absence of a static magnetic field. Li et al. [22] used endothelial progenitor cells loaded with SPIO as well as similar methods and magnet strength to deliver cells following brain infarction. 

SPIO was reported to have low toxicity in the human body [23]. Iron oxide nanoparticles are also well-known to be harmless and non-cytotoxic under 100 μg/mL. Ankamwar et al. [24] reported that the toxicity of Fe_3_O_4_ NPs coated with tetramethylammonium 11-aminoundecanoate was concentration-dependent, showing non-toxicity over the concentration range of 0.1–10 μg/mL but cytotoxicity at 100 μg/mL. Shen et al. [13] reported no negative effects on hNPC viability, proliferation, and differentiation following labeling with MIRB. In this study, we found that magnetized MSCs under 15 μg/mL IRB at 24 h showed similar viability as non-labeled MSCs in vitro. Despite the presence of a static magnetic field, MSCs-IRBs showed normal viability, proliferation, and differentiation properties in vivo and in vitro.

Upon internalization into cells, SPIO can induce toxicity by generating ROS [14,15]. SPIO may be degraded into iron ions within lysosomes by hydrolyzing enzymes. This free iron can react with hydrogen peroxide and oxygen produced by the mitochondria after crossing the mitochondrial membrane. Therefore, iron overload from SPIO exposure may result in harmful cellular consequences, finally leading to cell death. A study demonstrated that the ROS produced by iron-nanoparticles induced GSK-3b (Glycogen synthase kinase 3β) inhibition by activating the Akt signaling pathway, altering actin dynamics such as cell migration [25]. Another study investigating the toxic effect of Ferucarbotran (Resovist) revealed that MSCs showed enhanced cell proliferation with changes in the expression of cell cycle control genes and a reduction in intracellular hydrogen peroxide [26]. One approach for improving the homing capacity of MSCs is to culture MSCs under hypoxic conditions. At 15 μg/mL IRB for 24 h in vitro, the thresholds of ROS in magnetized MSCs was increased by nearly 2-fold. The stimulated ROS system may help in the homing of magnetized MSCs to increase the expression of CXCR4 on the cell surface. Therefore, this result demonstrates that adding IRB to MSCs can increase homing without a magnetic field.

The CXCR4-SDF-1 axis is known as an important factor in bone marrow homing [27]. Overexpression of CXCR4 in MSCs by internalization of nanoparticles can increase in vivo homing of MSCs into ischemic areas of the myocardium [28]. Insulin-like growth factor-1 treatment of rat MSCs was revealed to increase MSC migration in response to SDF-1 via CXCR4 receptor signaling. The SDF-1/CXCR4 axis may also contribute to the migration of MSCs into the brain and towards glioma tissue in irradiated animals [29]. Induction of CXCR4 expression by a simple method in injured tissue, which increased the expression of SDF-1, suggests that iron oxide nanoparticles can be used for internalization, and iron oxide is expected to be useful for biological applications.

Intranasal delivery of MSCs to the olfactory bulb appears to be a promising approach for the therapy of central nervous system diseases. Recent studies reported the nasal system as a novel stem cell delivery route to the brain [30,31,32]. MSCs transported into the nasal cavity have been shown to migrate through the cribriform plate and into brain tissue via the olfactory and trigeminal pathways. Lusine et al. demonstrated that noninvasive intranasal delivery of MSCs is a promising approach for the therapeutic treatment of Parkinson’s disease. The olfactory system has been widely applied as a model in studies of neural regeneration and axon rewiring [33]. Wounds to olfactory cells in the neuroepithelium are not reversible following damage of the basal cell layer, but when spared, regenerated basal cells lead to reconstruction of the sensory epithelium and subsequent restoration of olfactory function [9]. Therefore, intranasal delivery of magnetized MSCs in an injured olfactory mouse model is an effective method of demonstrating the homing phenomenon of MSCs using nanoparticles.

## 4. Materials and Methods

### 4.1. Characterization of NPs

Iron oxide nanoparticles with rhodamine B (IRBs) were obtained from Ocean Nanotech. The size and charge properties of IRBs were measured by TEM and with a Zetasizer-ZS90 (Malvern Instruments Ltd., Malvern, UK). IRBs were well-dispersed in an aqueous solution. For TEM analysis, after 10-min bath sonication, 2 μL IRBs were dropped on the carbon grid three times and completely dried. IRBs were observed by TEM (JEM-ARM 200F, JEOL, Tokyo, Japan). The diameter of IRBs was analyzed with ImageJ software (NIH, Bethesda, MD, USA). To obtain the ζ-potential of IRBs, 20 μL IRBs and 980 μL distilled water were mixed into a folded capillary cell (Malvern Instruments) after 10-min bath sonication.

### 4.2. Cell Culture

GFP-labeled MSCs derived from C57BL/6 mouse tibia bone marrow were obtained from Cyagen Biosciences, Inc. (cat. MUBMX-01101; Santa Clara, CA, USA), cultured in low-glucose DMEM (Hyclone, Logan, UT, USA) containing 2 ng/mL of basic fibroblast growth factor and 10% fetal bovine serum (Gibco, Grand Island, NY, USA), and maintained at 37 °C in a humidified atmosphere of 5% CO_2_. The cell culture medium was replaced every three days, and bone marrow-derived MSCs were collected by trypsin (0.25%, Invitrogen, Carlsbad, CA, USA) digestion. All experiments were performed using MSCs at 3–5 passages.

### 4.3. Internalizing Iron Oxide Nanoparticles with Rhodamine B into MSCs

GFP-labeled MSCs were seeded onto confocal dishes (SPL Life Sciences, Gyeonggi-do, Korea) at 1 × 10^5^ cells per well. After 24 h incubation, the culture media was exchanged with 2 mL of fresh media and cells were incubated for 0, 3, 6, and 24 h with 15 μg/mL IRBs. The MSCs were washed twice using PBS, fixed in 10% formalin, and stained with DAPI. Images were captured with a fluorescence microscope (Eclipse Ti-U, Nikon, Tokyo, Japan) and further analyzed to confirm the intracellular localization and quantity of IRBs. To quantify the internalization ratio in MSCs, all MSCs were counted in the fluorescence images. The number of RhB-positive cells was divided by the total number of cells.

### 4.4. Cytotoxicity of IRB-MSCs

MSCs were seeded into 96-well surface-treated plates at a density of 5 × 10^3^ cells per well and incubated at 37 °C and 5% CO_2_ for 24 h. The culture media were replaced with 100 μL of fresh media containing different concentrations of Fe in IRBs. Cells without IRBs were measured as a control. After 24 h of incubation, the cells were washed and incubated in cell culture media. In vitro cytotoxicity was evaluated by using the CCK-8 (Cell Counting Kit-8). For this assay, 10 μL CCK-8 was added to each well under light protection. After 1 h incubation, absorbance values at 450 nm were measured with a multimode reader (Synergy HTX, BioTek, Winooski, VT, USA).

### 4.5. Magnetic Field Effects on Magnetized MSCs In Vitro

To estimate the homing effect of MSCs using IRBs, magnetic flux properties were evaluated by both simulation (COMSOL Multiphysics software, Stockholm, Sweden) and a Tesla meter (TM-701, KANETEC) using a 5 × 5 × 5 mm permanent regular hexahedron neodymium magnet. From the magnet, the magnetic pole surface showing north and south was used to attract the IRBs. MSCs on 6-well plates at a density of 1 × 10^5^ per well were incubated for 3 h. Fluorescence images were captured with a fluorescence microscope. MSCs were treated for 12 h with 15 μg/mL IRBs. Images were captured to determine whether IRBs were internalized into the MSCs. Floating MSCs were incubated with the magnet for 24 h on six-well plates at a density of 1 × 10^5^ per well and MSCs guided by the magnet were confirmed using a fluorescence microscope.

### 4.6. Analysis of Real-Time PCR

Total RNA was extracted from the magnetized MSCs in vitro to evaluate CXCR4 expression. In an animal study, total RNA was collected using TRIzol Reagent (Invitrogen) from the sensory epithelium of both olfactory bulbs of three mice, which were sacrificed seven days after treatment. Total RNA was subjected to reverse transcription using SYBR^®^ Select master mix (Applied Biosystems, Foster City, CA, USA). Real-time RT-PCR was performed using the Applied Biosystem’s sequence detection system 7900 to quantify SDF-1 levels. The following primers were used for sequencing: CXCR4, forward: 5′-CAG CAT CGA CTC CTT CAT CC-3′ and reverse: 5′-GGT TCA GGC AAC AGT GGA AG-3′ (119 base pairs), SDF-1, forward: 5′-CGC CAG AGC CAA CGT CAA GC-3′ and reverse: 5′-TTT GGG TCA ATG CAC ACT TG-3′; β-actin, forward: 5′-CGT GCG TGA CAT CCA AGA GAA-3′ and reverse: 5′-TGG ATG CCA CAG GAT TCC AT-3′. To detect the possibility of genomic DNA amplification, no-template controls were utilized and allowed when the *C*_t_ value was at least nine cycles greater than the template run. Duplicate measurements were performed and accepted if the difference in *C*_t_ values between the duplicates was less than 1. Real-time PCR data were normalized to the level of β-actin, and the relative quantity of mRNA was determined using the comparative cycle threshold method.

### 4.7. Western Blotting and Fluorescence-Activated Cell Sorting

MSCs were incubated with IRBs for 2 h and then washed twice with PBS. Fresh medium was utilized for different time periods. After collecting the cells, CXCR4 expression of MSCs was evaluated by fluorescence-activated cell sorting and western blotting.

Cells (>10^5^) and olfactory tissue were lysed in Laemmli buffer according to the manufacturer’s instructions (Invitrogen) and separated on a 12% sodium dodecyl sulfate polyacrylamide gel. Membranes were hybridized with rabbit anti-human CXCR4 at 1:1000 (sc-9046; Santa Cruz Biotechnology, Dallas, TX, USA), with SDF-1 (ab18919; Abcam, Cambridge, UK), or anti-β-actin (1:500; Sigma, St. Louis, MO, USA). Anti-rabbit horseradish peroxidase secondary antibody was used at 1:1000 (Dako, Glostrup, Denmark). Deglycosylation was carried out using *N*-glycosidase F according to the manufacturer’s instructions (New England Biolabs, Ipswich, MA, USA).

To analyze CXCR4 expression in different subcellular protein fractions, MSCs were incubated with IRBs (108 μM) for 2 h. The cells were cultured for 22 h and the medium was replaced. After cell collection, the proteins expressed in cytoplasm, membrane, and nucleus were separated with a subcellular protein fractionation kit (Thermo Scientific, Waltham, MA, USA).

### 4.8. ROS Analysis

In vitro ROS analysis was conducted in 96-well surface-treated plates. MSCs were seeded at a density of 5 × 10^3^ cells per well and incubated at 37 °C and 5% CO_2_ for 24 h. After incubation, the cells were washed with PBS and 100 μL of fresh media containing different Fe concentrations in IRBs was added. Control groups did not contain added IRBs. After 24 h of incubation, MSCs were washed twice with PBS and incubated in fresh media and 10 μL 0.2 mM H_2_DCFDA PBS solution in the dark. Fluorescence values at 485 nm excitation/528 nm emission were measured with a multimode reader at each time point (0, 3, 6, and 24 h).

### 4.9. In Vivo Injection of Magnetized MSCs into Olfactory Injury Mouse Models

Twenty-four male C57BL/6 mice, including six control mice for the olfactory-injured model, were allowed free access to water and a regular mouse diet and kept at room temperature under a standard 12-h light/dark cycle for one week of acclimatization before the experiments. The animals were five weeks old and weighed approximately 18–25 g. Mice were anesthetized by intraperitoneal injection of 30 mg/kg tiletamine-zolazepam (Zoletil, 500 mg/vial; Virbac, Carros, France) and 10 mg/kg xylazine (Rompun; Bayer Korea, Ansan, Korea) and sacrificed by decapitation. Animal experiments were conducted in accordance with the guidelines of the Institutional Animal Care and Use Committee of Yonsei University, Korea (YWC-170721-1, 27 February 2017).

### 4.10. Immunofluorescence Staining in Olfactory Bulb

Normal and olfactory-injured mice were sacrificed after seven days of magnetized MSC injection. After cardiac perfusion, the skull bones were removed. Next, both olfactory bulbs were immersed in 4% paraformaldehyde (Biosesang Seongnam, Korea) in fixative for 24 h at 4 °C. The samples were embedded in optimal cutting temperature compound (Leica, Wetzlar, Germany) and sectioned at 2–10 μm thickness using a cryostat (Leica CM1850 Cryostat; Leica). A standard hematoxylin and eosin staining protocol was followed, with 1–3 min incubation in hematoxylin and 30–60 s staining with eosin, before mounting the samples.

Immunohistochemistry for SDF-1 was performed on cryosections of the cochlea in each group. Slide samples were incubated with appropriate primary antibodies as follows. An antibody against SDF-1 1 (cat# ab18919; Abcam) was used. Sections were incubated with the primary antibody overnight at 4 °C. After washing three times with 0.1 M PBS, the sections were incubated with an appropriate biotin-tagged secondary antibody at room temperature for 1 h. The sections were incubated in an avidin–biotin–peroxidase complex solution (Vector Laboratories, Inc., Burlingame, CA, USA) and developed with a diaminobenzidine substrate kit (Vector Laboratories, Inc.) after washing three times with 0.1 M PBS. The sections were dehydrated, mounted, and visualized with a BX50 microscope (Olympus, Tokyo, Japan), and digital images were captured.

### 4.11. Statistical Analysis

Statistical analysis was performed using SPSS statistical package version 17.0 (SPSS, Inc., Chicago, IL, USA). Descriptive results of continuous variables are expressed as the mean ± standard deviation (SD) for normally distributed variables. Means were compared by two-way analysis of variance. The level of statistical significance was set to 0.05.

## 5. Conclusions

Our study demonstrated that noninvasive intranasal delivery of magnetized MSCs with IRBs is a promising method for treating an olfactory-injured mouse model. Using complementary methods, we demonstrated the following: (i) migration of magnetized MSCs with IRBs under a magnetic field in vitro; (ii) increase in CXCR4 by internalizing IRBs into MSCs; and (iii) improvement in homing of magnetized MSCs into the injured olfactory epithelium in vivo. We are currently assessing the long-term effects (regeneration and cytotoxicity) of magnetized MSCs in olfactory-injured mice with different magnetic fields, nanoparticle concentrations, and nanoparticle shapes in MSCs.

## Supplementary Materials

The following are available online at https://zenodo.org/record/1243310#.WvEGxlKSCJ0.

## Abbreviations

MSCs	Mesenchymal stem cells
SPIO	Superparamagnetic iron oxide
IRBs	SPIO nanoparticles with rhodamine b
ROS	Reactive oxygen species
CXCR4	C-X-C chemokine receptor type 4
SDF-1	Stromal cell-derived factor 1
GFP	Green fluorescent protein
