# Supplementary material for: Enhanced Homing Technique of Mesenchymal Stem Cells Using Iron Oxide Nanoparticles by Magnetic Attraction in Olfactory-Injured Mouse Models
Source: Int J Mol Sci. 2018 May 5;19(5):1376. doi: 10.3390/ijms19051376 (PMC5983763; doi:10.3390/ijms19051376)
Supplement: Supplementary file 2 [file ijms-19-01376-s002.pdf]

International Journal of
Molecular Sciences

MDPI

Supplementary

Enhanced Homing Technique of Mesenchymal Stem Cells Using Iron Oxide Nanoparticles by Magnetic Attraction in Olfactory-Injured Mouse Models

Wan Su Yun [4,†], Jin Sil Choi [1,2,†], Hyun Mi Ju [1,2], Min Hee Kim [1,2], Seong Jin Choi [3], Eun Seol Oh [4], Young Joon Seo [1,2,*] and Jaehong Key [4,*]

[1] Laboratory of Smile Snail, Yonsei University Wonju College of Medicine, Wonju, Korea
[2] Department of Otorhinolaryngology, Yonsei University Wonju College of Medicine, Wonju, Korea; towtowtow92@naver.com (J.S.C.); skdi1082@naver.com (H.M.J.); mini.aclice11234@gmail.com (M.H.K.)
[3] Department of Obstetrics and Gynecology, Yonsei University Wonju College of Medicine, Wonju, Korea; choisj@yonsei.ac.kr
[4] Department of Biomedical Engineering, Yonsei University Wonju College of Medicine, Wonju, Korea; ip9801@naver.com (W.S.Y.); marchman10@hanmail.net (E.S.O.)
* Correspondence: okas2000@hanmail.net (Y.J.S.); jkey@yonsei.ac.kr (J.K.); Tel.: +82-33-741-0644 (Y.J.S.); +82-33-760-2857 (J.K.)
† Contributed equally to this work.

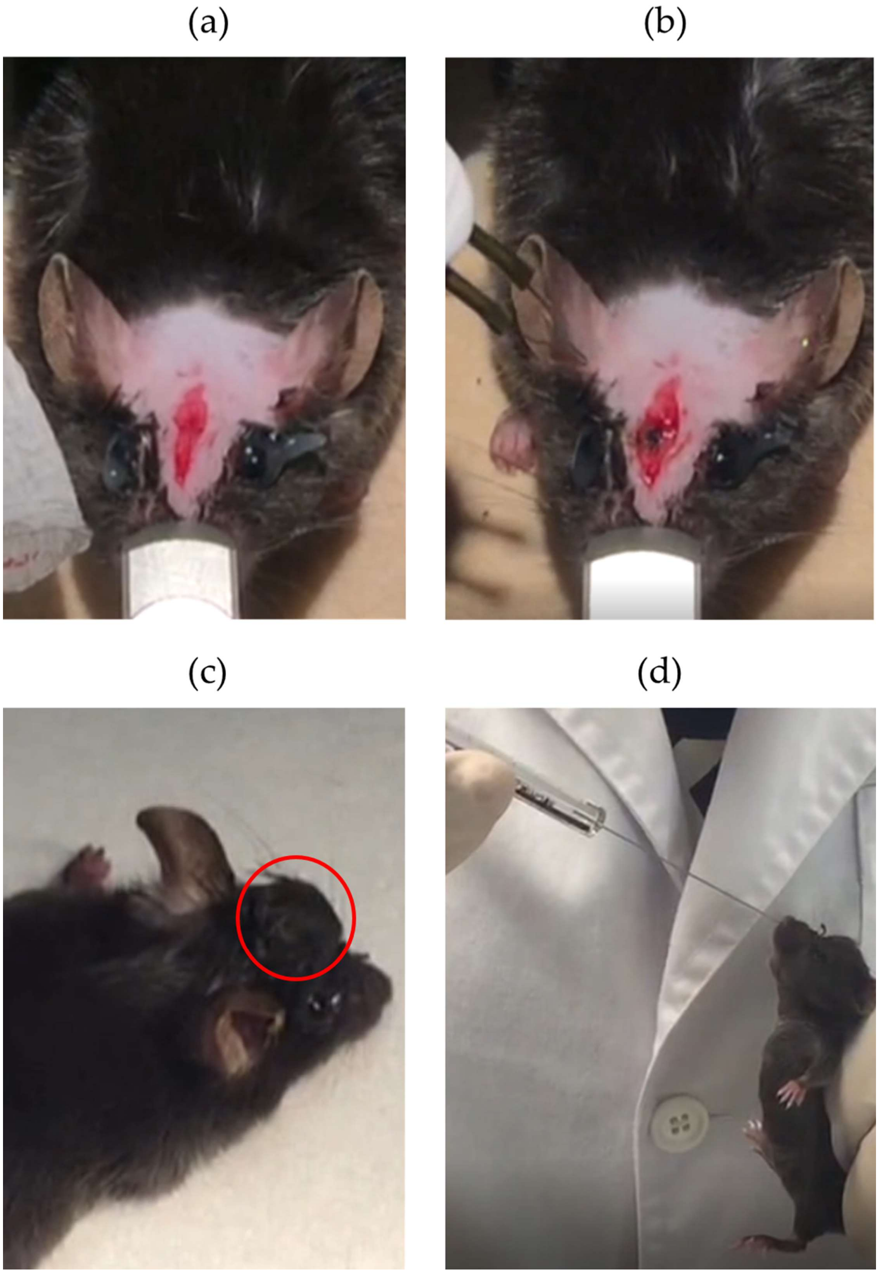

Figure S1. Procedures for migration of magnetized MSCs with IRBs in olfactory-injured mouse models. (**a**) After exposure of the scalp bone to remove the skin covering the skull with scissors, a 2-mm cranial window was opened on the exposed bone. (**b**) Bipolar coagulation was used with a 1-mm depth for 100 ms on only one side of the olfactory bulb. (**c**) The 5-mm cuboidal magnet was inserted into the scalp, and then the scalp was sutured. (**d**) Injection of magnetized MSCs into olfactory-injured mouse models was performed with a 50-μL Hamilton syringe via each nostril.

Damaged cells in the olfactory mouse model were confirmed by H&E staining and immunohistochemistry results of SDF-1 chemokines secreted from the damaged cells (Figure S2). H&E staining results clearly revealed the destructed left olfactory bulb compared to the right bulb (Figure S2A). The damaged site also showed high secretion of SDF-1 in immunohistochemistry analysis (Figure S2B). This showed that the olfactory mouse model was well-prepared.

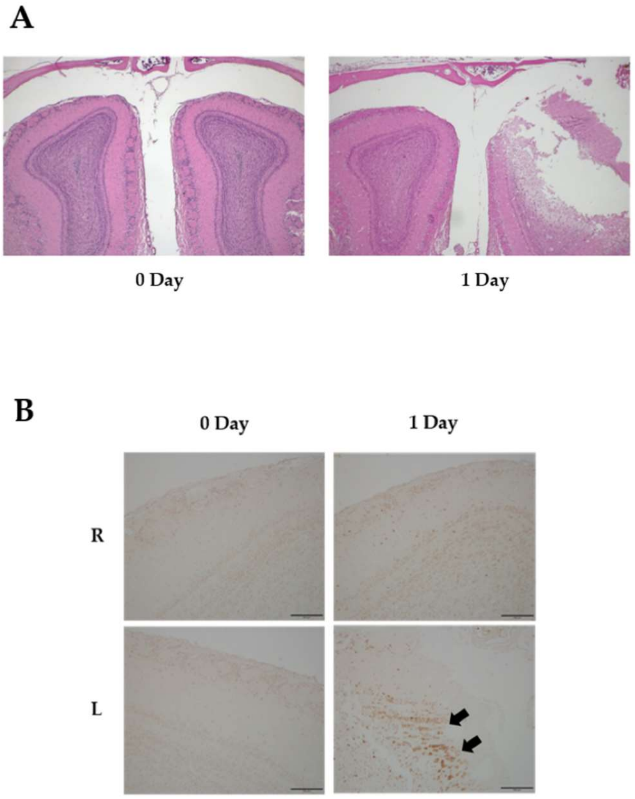

Figure S2. H&E staining and SDF-1 expression in olfactory injured mouse models. (**A**) Destruction of left olfactory bulbs after one day was observed via H&E staining. (**B**) Distribution of SDF-1 chemokines (black arrows) secreted from damaged cells around the injured area was confirmed by immunohistochemistry.
